# Contrast-Enhanced Magnetic Resonance Imaging Based T1 Mapping and Extracellular Volume Fractions Are Associated with Peripheral Artery Disease

**DOI:** 10.3390/jcdd11060181

**Published:** 2024-06-14

**Authors:** Asem I. Fitian, Michael C. Shieh, Olga A. Gimnich, Tatiana Belousova, Addison A. Taylor, Christie M. Ballantyne, Jean Bismuth, Dipan J. Shah, Gerd Brunner

**Affiliations:** 1 Penn State Heart and Vascular Institute, College of Medicine, Pennsylvania State University, Hershey, PA 17033, USA; 2 Section of Cardiovascular Research, Department of Medicine, Baylor College of Medicine, Houston, TX 77030, USA; 3 Methodist DeBakey Heart and Vascular Center, Houston Methodist Hospital, Houston, TX 77030, USA; 4 Michael E DeBakey VA Medical Center, Houston, TX 77030, USA; 5 Section of Cardiology, Department of Medicine, Baylor College of Medicine, Houston, TX 77030, USA; 6 Division of Vascular Surgery, University of South Florida Health Morsani School of Medicine, Tampa, FL 33620, USA

**Keywords:** peripheral artery disease, magnetic resonance imaging, T1 mapping, extracellular volume fraction, skeletal calf muscle

## Abstract

Background: Extracellular volume fraction (ECV), measured with contrast-enhanced magnetic resonance imaging (CE-MRI), has been utilized to study myocardial fibrosis, but its role in peripheral artery disease (PAD) remains unknown. We hypothesized that T1 mapping and ECV differ between PAD patients and matched controls. Methods and Results: A total of 37 individuals (18 PAD patients and 19 matched controls) underwent 3.0T CE-MRI. Skeletal calf muscle T1 mapping was performed before and after gadolinium contrast with a motion-corrected modified look–locker inversion recovery (MOLLI) pulse sequence. T1 values were calculated with a three-parameter Levenberg–Marquardt curve fitting algorithm. ECV and T1 maps were quantified in five calf muscle compartments (anterior [AM], lateral [LM], and deep posterior [DM] muscle groups; soleus [SM] and gastrocnemius [GM] muscles). Averaged peak blood pool T1 values were obtained from the posterior and anterior tibialis and peroneal arteries. T1 values and ECV are heterogeneous across calf muscle compartments. Native peak T1 values of the AM, LM, and DM were significantly higher in PAD patients compared to controls (all *p* < 0.028). ECVs of the AM and SM were significantly higher in PAD patients compared to controls (AM: 26.4% (21.2, 31.6) vs. 17.3% (10.2, 25.1), *p* = 0.046; SM: 22.7% (19.5, 27.8) vs. 13.8% (10.2, 19.1), *p* = 0.020). Conclusions: Native peak T1 values across all five calf muscle compartments, and ECV fractions of the anterior muscle group and the soleus muscle were significantly elevated in PAD patients compared with matched controls. Non-invasive T1 mapping and ECV quantification may be of interest for the study of PAD.

## 1. Introduction

Over 200 million adults globally are estimated to suffer from peripheral artery disease (PAD) [1,2]. PAD is associated with a high risk of cardiovascular mortality and morbidity [3,4]. Lower extremity blood flow is impaired in PAD patients and can manifest in intermittent claudication (IC), the primary symptom. The 10-year mortality rate of claudicants is approximately 50%, highlighting the importance of early disease detection to provide timely therapy [5,6,7]. The predominant location of claudication pain is in the skeletal calf muscles, and previous studies have reported associations between impaired contrast-enhanced magnetic resonance imaging (CE-MRI) muscle perfusion in PAD patients with IC compared with matched controls [8,9,10].

Advances in magnetic resonance imaging (MRI) have provided avenues to quantify tissue characteristics. Myocardial extracellular volume fraction (ECV) quantification and T1 mapping have been reported on extensively [11,12]. T1 mapping has proven effective for detecting diffuse myocardial disease in the early stages [5,13]. Previous studies reported the high reproducibility of myocardial T1 mapping [14,15]. However, the role of T1 mapping and ECV values remains poorly understood in PAD [16]. For the present work, we studied the role of the T1 mapping and ECV quantification of the skeletal calf muscles in PAD patients and controls. We hypothesized that native peak T1 times and ECV measures are elevated in PAD patients compared with matched controls.

## 2. Materials and Methods

The study was approved by the local institutional review board and conducted in keeping with the Helsinki Declaration of 1973. Study participants provided informed consent. Study participants were recruited at the Houston Methodist Hospital and the Michael E. DeBakey Veterans Affairs Medical Center, Houston, TX, USA. PAD patients with lifestyle-limiting intermittent claudication and controls without PAD were recruited and matched on the basis of age, gender, and ethnicity. The standard of care continued uninterrupted throughout the study subject participation. Patients with contraindications to MRI and those with an estimated glomerular filtration rate (eGFR) ≤ 40 mL/min/1.73 m^2^ were excluded from the study.

PAD patients and matched controls underwent lower extremity CE-MRI utilizing a 36-element bilateral peripheral angio array coil on a 3.0T MRI system (Siemens, Magnetom Verio or Trio, Erlangen, Germany). Study participants were positioned supine on the table feet-first, and MR imaging was performed at the mid-calf level. Localizers of the lower extremities were acquired (field of view (FOV): 19.9 × 39.9 cm). Then, as reported before, calf muscle perfusion imaging was performed and post-reactive hyperemia was induced with bilateral MRI-compatible blood-pressure cuffs positioned above the knees (duration: 3.5 min, pressure: 170 mm Hg) [8,17]. CE-MRI perfusion scans were acquired after rapid cuff deflation and the administration of a gadolinium-based contrast agent (GBCA) with a high-resolution saturation recovery gradient echo (GRE) pulse sequence (repetition time [TR]/echo time [TE]: 2.7/1.23 ms; slice thickness [ST] = 10 mm; flip angle [FA] = 30°; FOV = 17.5 × 35.0 cm; matrix = 144 × 288, temporal resolution = 409 ms) [8,17]. T1 mapping was performed before and after administering a GBCA with a motion-corrected modified look–locker inversion recovery (MOLLI) pulse sequence. T1 MOLLI scans were acquired prior to cuff inflation with the following parameters: TR = 675 millisecond (ms), TE = 1.11 ms, FA = 35°, echo train length = 1, matrix = 128 × 256, FOV = 19.0 × 38.0 cm, inversion time (TI) = [90, 240, 756, 923, 1440, 2106, 2790] ms, ST = 6 mm, bandwidth = 1028 Hz/pixel, and an in-plane pixel resolution= 1.48 × 1.48 mm. The GBCA was administered intravenously (gadopentetate dimeglumine [Magnevist, Bayer Healthcare, Whippany, NJ, USA] at 0.2 mmol/kg, or gadobutrol [Gadavist, Bayer Healthcare], at 0.1 mmol/kg) with flow rates of 2–4 mL/s proceeded by a 20 mL saline flush. Post-contrast T1 mapping was performed after the completion of all contrast-enhanced perfusion sequences. All study participants underwent at least 3 sets of perfusion imaging scanning (500 frames each with a temporal resolution of 409 ms resulting in a minimum duration of 613.5 s). Therefore, the minimum delay time for T1 mapping after the contrast administration was over 10 min (613.5 s). Native T1 mapping was performed prior to blood pressure cuff inflation. Therefore, the minimum total delay time between native and post-contrast T1 mapping was over 13 min (blood pressure cuff inflation: 3.5 min (210 s) + perfusion imaging: 10.2 min (613.5 s) = 13.7 min). MRI scans were saved in the DICOM format prior to image analysis and additional details of this study have been reported previously [17].

The arterial lumens of the anterior tibialis (AT), posterior tibial (PT), and peroneal artery (PE) were segmented and visualized as permitted by contrast enhancement using Sante DICOM Editor Version 3.0 (Santesoft, Nicosia, Cyprus). The reproducibility and quality of the lumen frame analysis and interpretation were assessed through intra-observer reproducibility analyses. In this study, we did not perform inter-observer variability analysis. However, we have previously reported inter-reader reproducibility for the same patients and the same skeletal calf muscle anatomy (intra-class correlation (ICC) was excellent: 0.91 (0.62, 0.97), *n* = 20) [17]. Cross-sectional leg muscle area (CSLMA) was measured using our previously described methodology [8].

**Figure 1:**
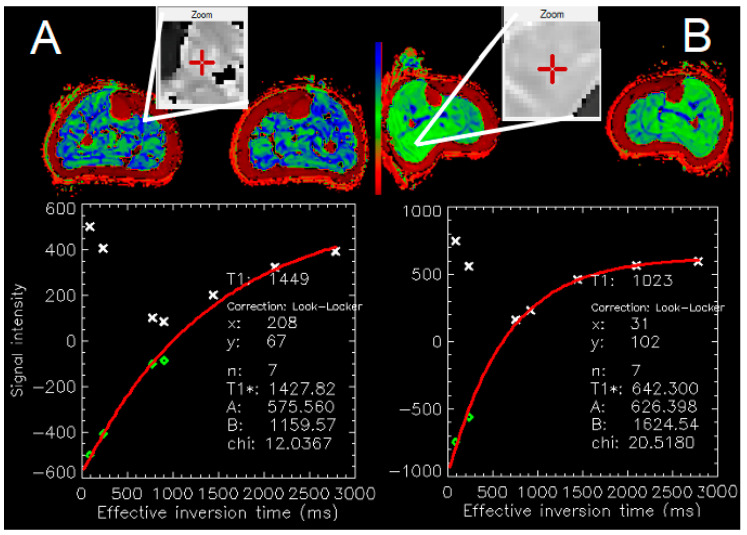
3T MRI MOLLI T1 map (mid-calf level). Panel (**A**): PAD patient. Zoom area from the soleus muscle. Panel (**B**): Matched control. Zoom area: soleus/gastrocnemius muscle. Note the significantly higher T1 values in the PAD patient (1449 ms) vs. control (1023 ms). MRMap [20], PRISM color-table.

T1 values are reported in ms and were obtained using MRmap version 1.4 (Congenital Heart Disease and Pediatric Cardiology, Deutsches Herzzentrum Berlin, Germany; Figure 1) [18,19,20]. The calculation of T1 values was based on a 3-parameter Levenberg–Marquardt curve fitting algorithm (y = A − B exp(−TI/T1), with A = scaling factor for the signal intensity, B = measure of the quality of the inversion.

We report peak T1 values across each of the five calf muscle compartments including the anterior (AM), lateral (LM), and deep posterior (DM) muscle groups; and the soleus (SM) and gastrocnemius (GM) muscles. Peak T1 values were calculated as the maximum T1 values for a given region of interest (ROI). We also reported mean T1 values, which were calculated by averaging the T1 values of all voxels in a given ROI. We also report the average peak T1 value, which was calculated by averaging the maximum T1 values over the five calf muscle compartments. Averaged peak blood pool T1 values were obtained from the posterior (PT) and anterior tibialis (AT) and peroneal artery (PE). The respective T1 values were calculated for pre-contrast (native) and post-contrast scans.

ECV was calculated for each muscle group as (1-hematocrit in %) × [(1/T1m post) − (1/T1m pre)]/[(1/T1b post) − (1/T1b pre)], where T1m denotes the maximum T1 value in ms of the respective muscle compartments and T1b in ms the averaged peak arterial blood T1 value at pre-contrast (pre), and post-contrast (post), respectively.

## 3. Statistical Analysis

Differences between PAD patients and matched controls for categorical variables were analyzed with the Chi-square or the Fisher exact tests. Normally distributed variables were analyzed with an independent sample Student’s t-test, while the Mann–Whitney–Wilcoxon test was used for non-normal variables. The Shapiro–Wilk test was utilized to determine variable normality. Associations between MRI-derived measures and markers of PAD were analyzed with univariate linear regression. Intra-observer variability was determined with the ICC coefficient using a two-way random-effects model. An ICC > 0.7 was considered as an excellent agreement [21,22,23,24]. All statistical tests were two-sided, and a *p*-value < 0.05 was used as the cutoff for statistical significance. The statistical analyses were performed with Stata Statistical Software (Release 13.1, StataCorp LP, College Station, TX, USA). MRI DICOM scans were analyzed with the Sante DICOM Editor (version 3.0, Santesoft, Nicosia, Cyprus), the ImageJ software (version 1.54d, National Institutes of Health, Bethesda, MD, USA), and MRmap version 1.4 (Deutsches Herzzentrum, Berlin, Germany).

## 4. Results

### 4.1. Patient Demographics

Patient demographic and clinical characteristics are shown in Table 1. Briefly, 66 individuals were enrolled, 5 participants did not complete baseline imaging, and an additional 24 participants were excluded due to poor image quality or incomplete MRI exams needed for the analysis. A total of 37 participants were included in the analysis (18 PAD patients and 19 matched controls). PAD patients and controls were similar in age, gender, and race. PAD patients compared to controls were more likely hypertensive and hyperlipidemic and had a higher rate of smoking history and prior lower extremity revascularization. As expected, PAD patients had a significantly lower ankle–brachial index (ABI), shorter peak walking time (PWT), and were less likely to complete 6 min of treadmill walking compared with matched controls.

### 4.2. Intra-Observer Reproducibility

Intra-observer reproducibility was excellent for arterial tracings, and the delineations of the leg and the AM, LM, DM, SM, and GM muscle compartments (all ICCs > 0.725; Table 2).

### 4.3. Skeletal Muscle Native Peak T1 Mapping

When averaged over all five calf muscle compartments (AM, LM, DM, SM, and GM), native peak T1 values were significantly higher in PAD patients when compared with matched controls (1902 (1877–1924) ms vs.1823 (1709–1883) ms, *p* = 0.005; Table 3). Native peak T1 values of the AM, LM, and DM were elevated in PAD patients compared with controls (all *p* < 0.03), while SM and GM did not differ (Table 3). Native minimum T1 times did not differ between calf muscle compartments.

### 4.4. Skeletal Muscle ECV

Skeletal muscle ECV values were elevated in PAD patients when compared to matched controls for the AM (26.4 (21.2–31.5) % vs. 17.3 (10.2–25.1) %, *p* = 0.046) and SM (22.7 (19.5–27.8) % vs. 13.8 (10.2–19.1) %, *p* = 0.020), while there was no difference in LM, DM, and GM (Table 3).

### 4.5. Native Peak T1 and ECV

Native peak T1, ECV values, and cross-sectional calf muscle compartment areas were compared among PAD patients and matched controls (Table 3). The composite averaged cross-sectional area of the calf muscles in PAD patients trended lower compared with the controls (*p* = 0.055). No significant differences were observed in T1 and ECV values of PAD patients with diabetes compared to those without (Supplementary Table S1).

### 4.6. Associations of ECV and Native Peak T1 Mapping with Clinical Measures of PAD

The pooled analysis of the study participants showed significant univariate associations between the ECV values of the DM and SM with the resting ABI but not with the AM, LM, and GM (Table 4). In addition, Supplementary Tables S2 and S3 show univariate associations for ECV values of each calf muscle compartment with clinical measures of PAD among controls and PAD patients, respectively.

The pooled analysis of the study participants showed a significant inverse univariate association between native peak T1 values of the AM and LM with PWT and the resting ABI, respectively (Table 5). Pooled univariate analysis for native peak T1 values averaged over all skeletal calf muscle compartments showed an inverse association with the resting ABI (*p* = 0.02; Table 6). Furthermore, a trend was observed for an inverse association between native peak T1 values averaged over all skeletal calf muscles with PWT (*p* = 0.09). In addition, Supplementary Tables S4 and S5 show univariate associations for native peak T1 values for all five calf muscles with clinical measures in PAD patients and controls, respectively. Supplementary Tables S6 (all not significant) and S7 show univariate associations for native peak T1 values averaged over all calf muscle compartments with clinical measures for PAD patients and controls, respectively.

When combining PAD patients and controls, only the native mean T1 value of the LM was inversely associated with the resting ABI (Supplementary Table S8). There were no significant associations between native mean T1 values and clinical markers when analyzed separately among PAD patients or controls, respectively (Supplementary Tables S9 and S10).

Pooled univariate linear regression analysis for ECV values averaged over all skeletal calf muscle compartments did not show any significant associations with markers of PAD (Table 7). Supplementary Tables S11 and S12 show univariate associations for mean ECV values averaged over all calf muscle compartments with clinical measures in PAD patients and controls, respectively (all not significant).

## 5. Discussion

In this study, we have performed quantitative tissue characterization utilizing T1 mapping and ECV assessment in patients with PAD and matched controls. We have reported three primary findings. The main finding is that native peak T1 times averaged over five skeletal calf muscle groups were higher in PAD patients compared with matched controls. Secondly, intra-observer reproducibility was excellent for arterial and skeletal calf muscle tracings. Thirdly, skeletal muscle ECV values were significantly elevated in PAD patients compared with matched controls for the anterior muscle group and the soleus muscle.

This study adds to the growing body of evidence that T1 mapping and ECV quantification are providing reproducible measures of tissue characterization including but not limited to diffuse fibrosis and other pathophysiological changes in response to inflammation, the hallmark of atherosclerosis and PAD [15,16,25].

Prior works have demonstrated the value of T1 mapping in coronary artery disease and left ventricular dysfunction. Myocardial native T1 mapping and ECV quantification have provided new insights into the understanding of associations between cardiomyopathy, muscular dystrophy, and myocardial fibrosis [26]. A recent study concluded that ECV and contrast-enhanced compared with native T1 mapping showed superior predictive performance to assess left ventricular remodeling, 6 months post-acute myocardial infarction [27]. Elevated myocardial ECV values, indicating diffuse myocardial fibrosis, have been shown to be associated with heart failure outcomes among patients without the presence of myocardial scarring [15]. In this study, skeletal muscle ECV values were significantly higher in PAD patients compared with matched controls for the anterior muscle group and the soleus muscle but not in the other calf muscle compartments. This finding reflects prior reports of heterogeneity of MRI-based measures across calf muscle compartments in PAD patients [8,28]. Previous reports have indicated regional heterogeneity in native T1 values, which agrees with our findings [29]. T1 mapping has been reported for the assessment of interstitial calf muscle fibrosis in PAD patients [30]. The authors found a significant inverse correlation between T1 values of the posterior calf compartment and the 6 min walking test in PAD patients, and T1 mapping was highly reproducible which is in agreement with our findings. In this study, we also found a significant inverse association between the native peak T1 value of the later muscle compartment with the ABI in PAD patients.

Tissue characterization utilizing MRI T1 mapping has also been performed in patients with liver disease [31,32,33]. Yoon et al. reported that native and post-contrast T1 times were significantly prolonged and correlated with increasing Child–Pugh scores, an established measure of liver disease severity [34]. The prognostic value of T1 mapping has been reported in patients with chronic liver disease [35]. The findings of this study and prior reports in the literature clearly show a broad utility of T1 mapping and ECV quantification across various pathologies and disease stages. Future growth in utilizing T1 mapping and ECV measures as novel imaging biomarkers is anticipated.

This observational imaging study has limitations. This is a secondary analysis of previously reported imaging data from PAD patients [17]. The small sample size of this work precludes the generalizability of its findings but does provide an early groundwork that future studies with larger patient cohorts can expound upon to further assess the role of native peak T1 and ECV values in PAD. Therefore, prospective studies, powered to determine the role of T1 mapping in PAD, are needed to confirm the presented findings. The obtained preliminary analyses will be supportive for adequately designing and powering future prospective studies. We enrolled PAD patients with lifestyle-limiting intermittent claudication and, therefore, our results may not be applicable to patients with critical limb ischemia. Post-contrast T1 mapping was performed after a reactive hyperemia protocol. Therefore, the reported post-contrast T1 times may differ when compared to measurements obtained at rest (without reactive hyperemia). In this study, when excluding the duration of the blood pressure cuff inflation, post-contrast delay times were at least 10 min, which is comparable to previous reports of approximately 11–12 min [36]. More studies are needed to determine the optimal delay time for post-contrast T1 mapping. Native T1 mapping was performed before cuff inflation and, therefore, the post-reactive hyperemia delay times were over 13 min. We did not assess inter-scan reproducibility and recommend future studies to perform these analyses. Chronic kidney disease is a common comorbidity in PAD patients and future studies will need to differentiate the value of native T1 mapping, which can be performed without a gadolinium-based contrast agent, as compared to ECV measurements that require contrast. Future studies are also needed to determine whether T1 mapping and ECV values are associated with calf muscle capillary density or muscle fiber loss in PAD patients.

In conclusion, native peak T1 values averaged over all five calf muscle compartments, and ECV fractions for the anterior muscle group and the soleus muscle, were significantly elevated in PAD patients compared with matched controls. Non-invasive T1 mapping and ECV quantification may be of interest for the study of PAD.

## Figures and Tables

**Table 1:** Baseline patient characteristics.

Variables	PAD Patients (*n* = 18)	Controls (*n* = 19)	*p*-Value
Age (years)	67.6 ± 9.10	65.4 ± 7.19	0.43
Males, *n* (%)	11 (61.1)	11 (57.9)	0.84
Black race, *n* (%)	4 (22.2)	4 (21.1)	0.86
Body mass index (kg/m^2^)	27.1 ± 3.85	28.2 ± 5.20	0.48
Resting ABI	0.734 ± 0.22	1.16 ± 0.087	<0.0001
Delta ABI	0.179 ± 0.18	0.110 ± 0.097	0.16
History of smoking, *n* (%)	16 (88.9)	8 (42.1)	0.006
Diabetes, *n* (%)	7 (38.9)	3 (15.8)	0.11
Hypertension, *n* (%)	17 (94.4)	11 (57.9)	0.010
Hyperlipidemia, *n* (%)	17 (94.4)	11 (57.9)	0.010
Heart rate (bpm)	68.6 ± 15.9	63.7 ± 6.89	0.24
Hematocrit (%)	38.5 ± 5.73	41.2 ± 3.68	0.14
eGFR (mL/min/1.73 m^2^)	71.8 ± 21.4	78.4 ± 18.1	0.36
Anticoagulation, *n* (%)	8 (44.4)	3 (15.8)	0.06
ACE inhibitor, *n* (%)	8 44.4)	5 (26.3)	0.25
Beta blocker, *n* (%)	9 (50.0)	7 (36.8)	0.42
Claudication onset time (s)	112.6 ± 102.8	N/A	<0.0001
Peak walking time (s)	298.2 ± 88.0	355.3 ± 20.6	0.009
Completed 6 min treadmill walking, *n* (%)	10 (58.8)	18 (94.7)	0.010
Cholesterol-lowering drug use, *n* (%)	15 (83.3)	8 (42.1)	0.010
Coronary artery disease, *n* (%)	8 (44.4)	4 (21.1)	0.13
Lower extremity revascularization history, *n* (%)	12 (66.7)	0 (0.0)	<0.0001
Family history of coronary heart disease, *n* (%)	11 (61.1)	8 (42.1)	0.25

Continuous variables are reported as means ± standard deviations. PAD: peripheral artery disease; BMI: body mass index; eGFR: estimated glomerular filtration rate; ABI: ankle–brachial index; ACE: angiotensin-converting enzyme. PAD patients: delta ABI, *n* = 17; post-treadmill ABI, *n* = 17; eGFR, *n* = 14; claudication onset time, *n* = 17; peak walking time, *n* = 17; completed 6 min treadmill, *n* = 17. matched controls: heart rate, *n* = 18; hematocrit, *n* = 14; eGFR, *n* = 18; anticoagulation, *n* = 18; coronary artery disease, *n* = 18. N/A in claudication onset time means that there was no claudication in the control group.

**Table 2:** Intra-observer variability as determined with the intra-class correlation (ICC) coefficient using a two-way model.

	**Intra-Observer ICC for the Anterior Muscle Group (Right Side)**	**Intra-Observer ICC for the** **Lateral Muscle Group (Right Side)**	**Intra-Observer ICC for the** **Deep Posterior Muscle Group (Right Side)**	**Intra-Observer ICC for the** **Soleus Muscle (Right Side)**	**Intra-Observer ICC for the** **Gastrocnemius Muscle (Right Side)**
Average ICC (95% CI)	0.946 (0.885–0.975)	0.883 (0.746–0.946)	0.728 (0.430–0.871)	0.903 (0.794–0.954)	0.919 (0.828–0.962)
	**Intra-observer ICC for the anterior muscle group (left side)**	**Intra-observer ICC for the** **lateral muscle group (left side)**	**Intra-observer ICC for the** **deep posterior muscle group (left side)**	**Intra-observer ICC for the** **soleus muscle (left side)**	**Intra-observer ICC for the** **gastrocnemius muscle (left side)**
Average ICC (95% CI)	0.955 (0.904–0.979)	0.933 (0.857–0.969)	0.828 (0.633–0.920)	0.961 (0.914–0.982)	0.892 (0.772–0.949)
	**Intra-observer ICC for the anterior muscle group (bilateral average)**	**Intra-observer ICC for the** **lateral muscle group (bilateral average)**	**Intra-observer ICC for the** **deep posterior muscle group (bilateral average)**	**Intra-observer ICC for the** **soleus muscle (bilateral average)**	**Intra-observer ICC for the** **gastrocnemius muscle (bilateral average)**
Average ICC (95% CI)	0.954 (0.902–0.978)	0.942 (0.877–0.973)	0.786 (0.548–0.899)	0.948 (0.889–0.975)	0.914 (0.819–0.960)
	**Intra-observer ICC for the cross-sectional leg area (bilateral average)**	**Intra-observer ICC for the** **anterior tibialis artery (right side)**	**Intra-observer ICC for the** **anterior tibialis artery (left side)**	**Intra-observer ICC for the** **posterior tibialis artery (right side)**	**Intra-observer ICC for the** **posterior tibialis artery (left side)**
Average ICC (95% CI)	0.961 (0.917–0.982)	0.992 (0.992–0.992)	0.988 (0.987–0.989)	0.959 (0.957–0.960)	0.986 (0.985–0.987)
	**Intra-observer ICC for the** **peroneal** **artery (right side)**		**Intra-observer ICC for the** **peroneal** **artery (left side)**		
Average ICC (95% CI)	0.934 (0.924–0.942)		0.955 (0.953–0.957)		

ICC and confidence interval were calculated using a two-way model for 26 cases. ICC: intra-class correlation; CI: confidence interval.

**Table 3:** MRI measurements for PAD patients and matched controls.

Variables	PAD Patients (*n* = 18)	Controls (*n* = 19)	*p*-Value
Native peak T1 of composite arteries (ms)	1729 (1679–1810)	1688 (1637–1756)	0.17
Cross-sectional area, anterior muscle group (mm^2^)	795 (678–980)	971 (802–1309)	0.045
Cross-sectional area, lateral muscle group (mm^2^)	454 (385–554)	597 (483–803)	0.007
Cross-sectional area, deep posterior muscle group (mm^2^)	677 (531–803)	624 (491–947)	1.00
Cross-sectional area, soleus muscle (mm^2^)	1459 (1235–1882)	1759 (1333–2322)	0.18
Cross-sectional area, gastrocnemius muscle (mm^2^)	1326 (1099–1800)	1649 (1204–2146)	0.11
Average cross-sectional area (mm^2^)	954 (828–1175)	1175 (954–1546)	0.055
Native peak T1, anterior muscle group (ms)	1835.5 (188)	1746 (163)	0.027
Native peak T1, lateral muscle group (ms)	1907 (65)	1755 (279)	0.002
Native peak T1, deep posterior muscle group (ms)	1917.5 (106)	1782 (296)	0.012
Native peak T1, soleus muscle (ms)	1930 (143)	1899 (164)	0.29
Native peak T1, gastrocnemius muscle (ms)	1945.5 (50)	1934 (160)	0.74
Minimum T1, anterior muscle group (ms)	878 (777–963)	878 (777–963)	0.53
Minimum T1, lateral muscle group (ms)	897 (818–955)	840 (718–926)	0.45
Minimum T1, deep posterior muscle group (ms)	729 (637–802)	712 (595–889)	0.87
Minimum T1, soleus muscle (ms)	728 (643–813)	823 (797–912)	0.024
Minimum T1, gastrocnemius muscle (ms)	730 (524–900)	651 (0–809)	0.55
Average cross-sectional native peak T1 (ms)	1902 (1877–1924)	1823 (1709–1883)	0.005
Average cross-sectional mean T1 (ms)	1218 (1143–1263)	1190 (1140–1258)	0.45
ECV, anterior muscle group (%)	26.4 (21.2–31.5)	17.3 (10.2–25.1)	0.046
ECV, lateral muscle group (%)	21.7 (15.1–31.2)	24.7 (19.6–38.5)	0.43
ECV, deep posterior muscle group (%)	29.0 (22.5–36.1)	24.1 (16.5–31.0)	0.19
ECV, soleus muscle (%)	22.7 (19.5–27.8)	13.8 (10.2–19.1)	0.020
ECV, gastrocnemius muscle (%)	21.8 (15.1–26.5)	16.8 (13.3–23.6)	0.38
ECV, averaged over 5 muscle compartments (%)	22.9 (21.0–27.5)	24.5 (20.7–28.0)	0.68

Values are reported as median (interquartile range, IQR); PAD: peripheral artery disease; ms: milliseconds; ECV: extracellular volume fraction. Composite denotes the T1 average for the peroneal, posterior tibialis, and anterior tibialis arteries combined. Average cross-sectional: average T1 times over all five muscle groups. ECV in controls (*n* = 12), ECV in PAD patients (*n* = 16).

**Table 4:** Pooled univariate linear regression analyses for ECV of skeletal calf muscle compartments with clinical markers of peripheral artery disease.

	Independent Variables	*n*	β	Standard Error	R^2^	Adjusted r^2^	*p*-Value
ECV, AM (%)	Resting ABI	28	0.243	9.086	0.06	0.02	0.21
	Δ ABI	27	−0.213	−23.030	0.05	0.05	0.28
	Claudication onset time (s)	27	0.150	0.018	0.02	−0.02	0.46
	Peak walking time (s)	27	−0.201	−0.035	0.04	0.002	0.31
	Body mass index (kg/m^2^)	27	−0.216	−0.501	0.05	0.01	0.27
	eGFR (mL/min/1.73 m^2^)	27	0.079	0.041	0.006	−0.04	0.72
ECV, LM (%)	Resting ABI	28	0.244	9.086	0.06	0.02	0.21
	Δ ABI	27	−0.213	−23.034	0.05	0.01	0.28
	Claudication onset time (s)	27	−0.081	−0.009	0.01	−0.03	0.69
	Peak walking time (s)	27	0.146	0.023	0.02	−0.02	0.47
	Body mass index (kg/m^2^)	28	−0.052	−0.116	0.003	−0.04	0.79
	eGFR (mL/min/1.73 m^2^)	23	−0.122	−0.064	0.02	−0.03	0.58
ECV, DM (%)	Resting ABI	28	0.389	3.197	0.15	0.12	0.041
	Δ ABI	27	0.080	4.055	0.01	−0.03	0.69
	Claudication onset time (s)	27	0.135	0.013	0.02	−0.02	0.50
	Peak walking time (s)	27	−0.192	−0.027	0.04	−0.002	0.34
	Body mass index (kg/m^2^)	28	−0.580	−1.120	0.34	0.31	0.001
	eGFR (mL/min/1.73 m^2^)	23	0.373	0.176	0.14	0.10	0.08
ECV, SM (%)	Resting ABI	28	0.403	1.580	0.16	0.13	0.034
	Δ ABI	27	−0.093	−9.920	0.01	−0.03	0.64
	Claudication onset time (s)	27	0.226	0.024	0.05	0.01	0.26
	Peak walking time (s)	27	0.029	0.004	0.001	−0.04	0.89
	Body mass index (kg/m^2^)	28	−0.247	0.394	0.06	0.02	0.21
	eGFR (mL/min/1.73 m^2^)	23	0.244	0.128	0.06	0.01	0.26
ECV, GM (%)	Resting ABI	28	0.104	0.669	0.01	−0.03	0.60
	Δ ABI	27	0.140	5.410	0.02	−0.02	0.49
	Claudication onset time (s)	27	0.112	0.015	0.01	−0.03	0.58
	Peak walking time (s)	27	−0.022	−0.002	0.001	−0.04	0.91
	Body mass index (kg/m^2^)	28	−0.247	−0.511	0.06	0.02	0.21
	eGFR (mL/min/1.73 m^2^)	23	−0.020	0.007	0.00	−0.05	0.93

ECV: extracellular volume fraction; ABI: ankle–brachial index; Δ ABI: delta ABI; eGFR: estimated glomerular filtration rate. *n*: number of study subjects. AM: anterior muscle group. LM: lateral muscle group. DM: deep muscle group. SM: soleus muscle. GM: gastrocnemius muscle.

**Table 5:** Pooled univariate linear regression analyses for native peak T1 values of skeletal calf muscle compartments with clinical markers of peripheral artery disease.

	Independent Variables	*n*	β	Standard Error	R^2^	Adjusted r^2^	*p*-Value
Native peak T1 of AM (ms)	Resting ABI	37	−0.276	−73.6	0.08	0.05	0.10
	Δ ABI	36	0.201	237.4	0.04	0.01	0.24
	Claudication onset time (s)	36	0.083	0.159	0.01	−0.02	0.63
	Peak walking time (s)	36	−0.354	−0.893	0.13	0.10	0.03
	Body mass index (kg/m^2^)	37	0.153	5.79	0.02	−0.01	0.37
	eGFR (mL/min/1.73 m^2^)	31	−0.152	−1.33	0.02	−0.01	0.42
Native peak T1 of LM (ms)	Resting ABI	37	−0.359	−120.2	0.13	0.10	0.029
	Δ ABI	36	0.277	258.7	0.08	0.05	0.10
	Claudication onset time (s)	36	0.344	0.517	0.12	0.09	0.040
	Peak walking time (s)	36	−0.141	−0.281	0.02	−0.01	0.41
	Body mass index (kg/m^2^)	37	0.166	4.87	0.03	0.00	0.33
	eGFR (mL/min/1.73 m^2^)	31	0.049	0.347	0.002	−0.03	0.79
Native peak T1 of DM (ms)	Resting ABI	37	−0.102	−26.2	0.01	−0.02	0.55
	Δ ABI	36	0.216	240.8	0.05	0.02	0.21
	Claudication onset time (s)	36	−0.026	−0.046	0.00	−0.03	0.88
	Peak walking time (s)	36	−0.199	−0.472	0.04	0.01	0.25
	Body mass index (kg/m^2^)	37	0.110	3.85	0.01	−0.02	0.52
	eGFR (mL/min/1.73 m^2^)	31	0.011	0.093	0.00	−0.03	0.95
Native peak T1 of SM (ms)	Resting ABI	37	−0.141	−59.9	0.02	−0.01	0.41
	Δ ABI	36	0.192	153.0	0.04	0.01	0.26
	Claudication onset time (s)	36	0.122	0.156	0.02	−0.01	0.48
	Peak walking time (s)	36	−0.233	−0.395	0.05	0.03	0.17
	Body mass index (kg/m^2^)	37	−0.071	−1.78	0.01	−0.02	0.68
	eGFR (mL/min/1.73 m^2^)	31	−0.144	0.902	0.02	−0.01	0.44
Native peak T1 of GM (ms)	Resting ABI	37	−0.199	−75.8	0.04	0.01	0.24
	Δ ABI	36	0.185	131.7	0.03	0.01	0.28
	Claudication onset time (s)	36	0.024	0.027	0.001	−0.03	0.89
	Peak walking time (s)	36	−0.091	−0.139	0.01	−0.02	0.60
	Body mass index (kg/m^2^)	37	0.353	7.94	0.12	0.10	0.032
	eGFR (mL/min/1.73 m^2^)	31	0.131	0.672	0.02	−0.02	0.48

ABI: ankle–brachial index; Δ ABI: difference between resting ABI and post-treadmill walking ABI; eGFR: estimated glomerular filtration rate; ms: milliseconds. AM: anterior muscle group. LM: lateral muscle group. DM: deep muscle group. SM: soleus muscle. GM: gastrocnemius muscle.

**Table 6:** Pooled univariate linear regression analysis for native peak T1 values averaged over all skeletal calf muscle compartments with clinical markers of peripheral artery disease.

	Independent Variables	*n*	β	Standard Error	R^2^	Adjusted r^2^	*p*-Value
Native peak T1 averaged over all calf muscle compartments (ms)	Resting ABI	37	−0.379	−145.1	0.14	0.12	0.021
	Δ ABI	36	0.289	204.3	0.08	0.06	0.09
	Claudication onset time (s)	36	0.143	0.163	0.02	−0.01	0.40
	Peak walking time (s)	36	−0.289	−0.436	0.08	0.06	0.09
	Body mass index (kg/m^2^)	37	0.183	4.14	0.03	0.01	0.28
	eGFR (mL/min/1.73 m^2^)						

ABI: ankle-brachial index; Δ ABI: difference between resting ABI and post-treadmill walking ABI; eGFR: estimated glomerular filtration rate; ms: milliseconds.

**Table 7:** Pooled univariate linear regression analysis for ECV values averaged over all skeletal calf muscle compartments with clinical markers of peripheral artery disease.

	Independent Variables	*n*	β	Standard Error	R^2^	Adjusted r^2^	*p*-Value
Mean ECV (averaged over all calf muscle compartments) (%)	Resting ABI	28	0.016	0.324	<0.001	−0.04	0.93
	Δ ABI	27	0.019	1.06	<0.001	−0.04	0.93
	Claudication onset time (s)	27	0.117	0.007	0.01	−0.03	0.56
	Peak walking time (s)	27	0.014	0.001	<0.001	−0.04	0.95
	Body mass index (kg/m^2^)	28	−0.091	−0.103	0.008	−0.03	0.65
	eGFR (mL/min/1.73 m^2^)	23	−0.058	−0.016	0.003	−0.04	0.79

ABI: ankle–brachial index; Δ ABI: difference between resting ABI and post-treadmill walking ABI; eGFR: estimated glomerular filtration rate; ECV: extracellular volume fraction.

## Data Availability

Data are contained within the article and Supplementary Materials.

## Supplementary Materials

The following supporting information can be downloaded at https://www.mdpi.com/article/10.3390/jcdd11060181/s1: Tables S1–S12: Supporting Tables on T1 mapping and ECV analyses.
