# Supplementary material for: Contrast-Enhanced Magnetic Resonance Imaging Based T1 Mapping and Extracellular Volume Fractions Are Associated with Peripheral Artery Disease
Source: J Cardiovasc Dev Dis. 2024 Jun 14;11(6):181. doi: 10.3390/jcdd11060181 (PMC11203653; doi:10.3390/jcdd11060181)
Supplement: Supplementary file 1 [file jcdd-11-00181-s001.zip › jcdd-2921062-supplementary.pdf]

Title:

Contrast-Enhanced Magnetic Resonance Imaging Based T1 Mapping and Extracellular Volume Fractions are Associated with Peripheral Artery Disease

Supplementary Table S1. MRI measurements of diabetic and non-diabetic patients with peripheral artery disease.

Variables	Diabetic PAD Patients (n=7)	Non-Diabetic PAD Patients (n=11)	P-value
Native peak T1, anterior muscle group (ms)	1955 ± 204	1811 ± 138	0.15
Native peak T1, lateral muscle group (ms)	1897 ± 134	1921 ± 136	0.89
Native peak T1, deep posterior muscle group (ms)	1903 ± 48	1916 ± 80	0.19
Native peak T1, soleus muscle (ms)	1980 ± 250	1917 ± 127	0.26
Native peak T1, gastrocnemius muscle (ms)	1947 ± 125	1932 ± 50	0.62
Average cross-sectional, native peak T1 (ms)	1924 ± 83	1911 ± 39	0.14
Average cross-sectional, mean T1 (ms)	1270 ± 138	1227 ± 84	0.49
ECV, anterior muscle group (%)	23.2 ± 13	28.1 ± 10	0.33
ECV, deep posterior muscle group (%)	28.5 ± 20	29.0 ± 10	0.91
ECV, lateral muscle group (%)	21.3 ± 16	23.1 ± 16	0.66
ECV, soleus muscle (%)	19.5 ± 9.1	24.6 ± 7.7	0.13
ECV, gastrocnemius muscle (%)	16.8 ± 14.9	22.7 ± 9.1	0.33
ECV, average cross-sectional (%)	26.8 ± 8.12	22.8 ± 2.5	0.33

PAD: peripheral artery disease; ms: milliseconds; ECV: extracellular volume fraction. Average cross-sectional: averaged T1 times or ECV over all five muscle groups. Diabetic PAD patients (n=6), Non-diabetic PAD patients (n=10).

Supplementary Table S2. Univariate linear regression analyses for ECV of skeletal calf muscle compartments with clinical markers of peripheral artery disease in matched controls.

	**Independent Variables**	***n***	**β**	**Standard Error**	**R^2**	**Adjusted r^2**	***p*-value**
ECV, AM (%)	Resting ABI	12	0.105	15.340	0.01	-0.09	0.74
	Δ ABI	12	0.463	327.50	0.22	0.14	0.13
	Claudication onset time (sec)	--	--	--	--	--	--
	Peak walking time (sec)	12	-0.326	123.690	0.11	0.02	0.30
	Body mass index (kg/m²)	12	0.023	0.038	0.001	-0.10	0.94
	eGFR (ml/min/1.73m²)	11	-0.120	-0.061	0.01	-0.09	0.73
ECV, LM (%)	Resting ABI	12	0.334	42.450	0.11	0.02	0.29
	Δ ABI	12	-0.246	-24.580	0.06	-0.03	0.44
	Claudication onset time (sec)	--	--	--	--	--	--
	Peak walking time (sec)	12	-0.326	-0.129	0.06	-0.03	0.44
	Body mass index (kg/m²)	12	0.023	0.038	0.001	-0.1	0.94
	eGFR (ml/min/1.73m²)	11	-0.560	-0.275	0.31	0.24	0.07
ECV, DM (%)	Resting ABI	12	0.134	17.270	0.02	-0.08	0.68
	Δ ABI	12	0.041	1.980	0.002	-0.10	0.90
	Claudication onset time (sec)	--	--	--	--	--	--
	Peak walking time (sec)	12	-0.350	-0.141	0.12	0.03	0.26
	Body mass index (kg/m²)	12	-0.726	-1.190	0.53	0.40	0.008
	eGFR (ml/min/1.73m²)	11	0.459	0.224	0.21	0.12	0.16
ECV, SM (%)	Resting ABI	12	0.0581	7.413	0.003	-0.09	0.86
	Δ ABI	12	0.066	6.617	0.004	-0.09	0.84
	Claudication onset time (sec)	--	--	--	--	--	--
	Peak walking time (sec)	12	0.045	0.017	0.004	-0.09	0.07
	Body mass index (kg/m²)	12	-0.610	0.476	0.14	0.05	0.23
	eGFR (ml/min/1.73m²)	11	0.173	0.087	0.03	-0.08	0.61
ECV, GM (%)	Resting ABI	12	0.066	7.210	0.004	-0.09	0.84
	Δ ABI	12	0.075	6.440	0.006	-0.09	0.82
	Claudication onset time (sec)	--	--	--	--	--	--
	Peak walking time (sec)	12	0.095	0.032	0.009	-0.09	0.77
	Body mass index (kg/m²)	12	0.335	0.464	0.11	0.02	0.29
	eGFR (ml/min/1.73m²)	11	-0.183	-0.076	0.03	-0.07	0.59

ECV: extracellular volume fraction; ABI: ankle-brachial index; Δ ABI: difference between resting ABI and post-treadmill walking ABI; eGFR: estimated glomerular filtration rate**.** AM: Anterior muscle group. LM: Lateral muscle group. DM: Deep muscle group. SM: Soleus muscle. GM: Gastrocnemius muscle.

Supplementary Table S3. Univariate linear regression analyses of patients with peripheral artery disease (PAD) for ECV of skeletal calf muscle compartments with clinical markers of PAD.

	**Independent Variables**	***n***	**β**	**Standard Error**	**R^2**	**Adjusted r^2**	***p*-value**
ECV, AM (%)	Resting ABI	16	0.332	0.194	0.15	0.07	0.21
	Δ ABI	15	-0.621	-66.7	0.39	0.34	0.013
	Claudication onset time (sec)	15	0.039	0.004	0.002	-0.07	0.89
	Peak walking time (sec)	15	-0.065	-0.008	0.004	-0.07	0.82
	Body mass index (kg/m²)	16	0.173	-0.480	0.03	-0.04	0.52
	eGFR (ml/min/1.73m²)	12	0.394	0.194	0.15	0.07	0.21
ECV, LM (%)	Resting ABI	16	0.115	5.480	0.01	-0.05	0.67
	Δ ABI	15	-0.143	-16.5	0.02	-0.05	0.61
	Claudication onset time (sec)	15	0.039	0.004	0.002	-0.05	0.89
	Peak walking time (sec)	15	0.195	0.026	0.04	-0.04	0.49
	Body mass index (kg/m²)	16	-0.242	-0.711	0.06	0.01	0.37
	eGFR (ml/min/1.73m²)	12	0.442	0.124	0.19	0.11	0.15
ECV, DM (%)	Resting ABI	16	0.332	12.5	0.11	0.05	0.21
	Δ ABI	15	-0.134	-12.0	0.02	-0.06	0.63
	Claudication onset time (sec)	15	-0.109	-0.009	0.01	-0.06	0.70
	Peak walking time (sec)	15	-0.033	-0.003	0.001	-0.08	0.91
	Body mass index (kg/m²)	16	-0.326	-0.758	0.10	0.04	0.22
	eGFR (ml/min/1.73m²)	12	0.442	0.193	0.19	0.12	0.15
ECV, SM (%)	Resting ABI	16	0.334	13.750	0.11	0.05	0.21
	Δ ABI	15	-0.407	-40.4	0.16	0.10	0.13
	Claudication onset time (sec)	15	-0.020	-0.002	0.00	-0.07	0.94
	Peak walking time (sec)	15	0.269	0.031	0.07	0.001	0.33
	Body mass index (kg/m²)	16	0.046	0.117	0.06	-0.01	0.87
	eGFR (ml/min/1.73m²)	12	0.474	0.238	0.23	0.15	0.12
ECV, GM (%)	Resting ABI	16	0.330	9.650	0.11	0.04	0.21
	Δ ABI	15	-0.260	-17.1	0.07	-0.004	0.35
	Claudication onset time (sec)	15	-0.009	-0.001	0.00	-0.08	0.97
	Peak walking time (sec)	15	0.051	0.004	0.003	-0.07	0.86
	Body mass index (kg/m²)	16	-0.059	-0.106	0.003	-0.07	0.83
	eGFR (ml/min/1.73m²)	12	0.257	0.087	0.11	0.04	0.42

ECV: extracellular volume fraction; ABI: ankle-brachial index; Δ ABI: difference between resting ABI and post-treadmill walking ABI; eGFR: estimated glomerular filtration rate**.** AM: Anterior muscle group. LM: Lateral muscle group. DM: Deep muscle group. SM: Soleus muscle. GM: Gastrocnemius muscle.

Supplementary Table S4. Univariate linear regression analysis for native peak T1 values of skeletal calf muscle compartments in patients with peripheral artery disease (PAD) with clinical markers of PAD.

	**Independent Variables**	***n***	**β**	**Standard Error**	**R^2**	**Adjusted r^2**	***p*-value**
Native peak T1 of AM (ms)	Resting ABI	18	-0.457	-165.7	0.21	0.16	0.06
	Δ ABI	17	-0.069	-31.1	0.005	-0.06	0.79
	Claudication onset time (sec)	17	-0.086	-0.068	0.007	-0.06	0.74
	Peak walking time (sec)	17	-0.084	-0.077	0.007	-0.06	0.75
	Body mass index (kg/m²)	18	0.090	1.85	0.008	-0.05	0.72
	eGFR (ml/min/1.73m²)	13	-0.076	-0.304	0.006	-0.09	0.81
Native peak T1 of LM (ms)	Resting ABI	18	0.098	133.1	0.01	-0.05	0.70
	Δ ABI	17	0.061	76.7	0.004	-0.06	0.82
	Claudication onset time (sec)	17	0.096	0.095	0.01	-0.06	0.71
	Peak walking time (sec)	17	-0.170	-0.441	0.03	-0.04	0.51
	Body mass index (kg/m²)	18	0.012	0.943	0.00	-0.06	0.96
	eGFR (ml/min/1.73m²)	13	-0.225	-0.966	0.05	-0.04	0.46
Native peak T1 of DM (ms)	Resting ABI	18	-0.543	-646.8	0.30	0.25	0.02
	Δ ABI	17	0.291	427.7	0.09	0.02	0.26
	Claudication onset time (sec)	17	-0.113	-0.082	0.01	-0.05	0.67
	Peak walking time (sec)	17	-0.033	-0.029	0.001	-0.07	0.90
	Body mass index (kg/m²)	18	-0.334	-6.810	0.11	0.06	0.18
	eGFR (ml/min/1.73m²)	13	-0.398	-5.480	0.16	0.08	0.18
Native peak T1 of SM (ms)	Resting ABI	18	-0.135	-64.80	0.02	-0.04	0.59
	Δ ABI	17	0.172	100.3	0.03	-0.04	0.51
	Claudication onset time (sec)	17	0.085	0.067	0.01	-0.06	0.75
	Peak walking time (sec)	17	-0.350	-0.418	0.12	0.06	0.17
	Body mass index (kg/m²)	18	0.226	6.15	0.05	-0.01	0.37
	eGFR (ml/min/1.73m²)	13	0.185	1.010	0.03	-0.05	0.54
Native peak T1 of GM (ms)	Resting ABI	18	0.029	10.5	.001	-0.06	0.91
	Δ ABI	17	-0.124	-53.7	0.02	-0.05	0.64
	Claudication onset time (sec)	17	-0.156	-0.119	0.03	-0.04	0.55
	Peak walking time (sec)	17	-0.140	-0.125	0.02	-0.05	0.59
	Body mass index (kg/m²)	18	0.147	2.99	0.02	-0.04	0.56
	eGFR (ml/min/1.73m²)	13	0.325	1.390	0.11	0.02	0.28

ABI: ankle-brachial index; Δ ABI: difference between resting ABI and post-treadmill walking ABI; eGFR: estimated glomerular filtration rate; ms: milliseconds**.** AM: Anterior muscle group. LM: Lateral muscle group. DM: Deep muscle group. SM: Soleus muscle. GM: Gastrocnemius muscle.

Supplementary Table S5. Univariate linear regression analyses for native peak T1 values of skeletal calf muscle compartments with clinical markers of peripheral artery disease in matched controls.

	**Independent Variables**	***n***	**β**	**Standard Error**	**R^2**	**Adjusted r^2**	***p*-value**
Native peak T1 of AM (ms)	Resting ABI	19	0.230	439.8	0.05	-0.003	0.34
	Δ ABI	19	0.013	22.3	0.00	-0.06	0.96
	Claudication onset time (sec)	--	--	--	--	--	--
	Peak walking time (sec)	19	0.101	0.822	0.01	-0.05	0.68
	Body mass index (kg/m²)	19	0.167	5.37	0.03	-0.03	0.49
	eGFR (ml/min/1.73m²)	18	-0.279	-2.66	0.08	0.02	0.26
Native peak T1 of LM (ms)	Resting ABI	19	-0.228	-381.9	0.05	-0.004	0.35
	Δ ABI	19	0.454	688.9	0.21	0.16	0.05
	Claudication onset time (sec)	--	--	--	--	--	--
	Peak walking time (sec)	19	0.145	1.03	0.02	-0.04	0.55
	Body mass index (kg/m²)	19	0.304	8.60	0.09	0.04	0.21
	eGFR (ml/min/1.73m²)	18	0.234	1.91	0.06	-0.004	0.35
Native peak T1 of DM (ms)	Resting ABI	19	-0.055	-106.3	0.003	-0.06	0.82
	Δ ABI	19	0.570	1006.3	0.33	0.29	0.011
	Claudication onset time (sec)	--	--	--	--	--	--
	Peak walking time (sec)	19	0.185	1.53	0.03	-0.02	0.45
	Body mass index (kg/m²)	19	0.205	6.72	0.04	-0.01	0.40
	eGFR (ml/min/1.73m²)	18	-0.012	-0.111	0.00	-0.06	0.96
Native peak T1 of SM (ms)	Resting ABI	19	0.172	244.0	0.03	-0.03	0.48
	Δ ABI	19	0.192	246.7	0.04	-0.02	0.43
	Claudication onset time (sec)	--	--	--	--	--	--
	Peak walking time (sec)	19	0.071	0.426	0.005	-0.05	0.77
	Body mass index (kg/m²)	19	-0.217	5.20	0.05	-0.01	0.37
	eGFR (ml/min/1.73m²)	18	0.151	1.07	0.02	-0.04	0.55
Native peak T1 of GM (ms)	Resting ABI	19	-0.418	-580.6	0.18	0.13	0.08
	Δ ABI	19	0.507	637.8	0.26	0.21	0.03
	Claudication onset time (sec)	--	--	--	--	--	--
	Peak walking time (sec)	19	0.159	0.939	0.03	-0.03	0.52
	Body mass index (kg/m²)	19	0.485	11.3	0.24	0.19	0.04
	eGFR (ml/min/1.73m²)	18	0.027	0.14	.001	-0.06	0.93

ABI: ankle-brachial index; Δ ABI: difference between resting ABI and post-treadmill walking ABI; eGFR: estimated glomerular filtration rate; ms: milliseconds**.** AM: Anterior muscle group. LM: Lateral muscle group. DM: Deep muscle group. SM: Soleus muscle. GM: Gastrocnemius muscle.

Supplementary Table S6. Univariate linear regression analysis for native peak T1 values averaged over all skeletal calf muscle compartments in patients with peripheral artery disease (PAD) with clinical markers of PAD.

	**Independent Variables**	***n***	**β**	**Standard Error**	**R^2**	**Adjusted r^2**	***p*-value**
Native peak T1 averaged over all calf muscle compartements (ms)	Resting ABI	18	-0.042	-14.5	0.002	-0.06	0.87
	Δ ABI	17	0.043	17.5	0.002	-0.07	0.87
	Claudication onset time (sec)	17	-0.339	-0.241	0.12	0.06	0.18
	Peak walking time (sec)	17	-0.325	-0.270	0.11	0.05	0.20
	Body mass index (kg/m²)	18	0.289	5.57	0.08	0.05	0.20
	eGFR (ml/min/1.73m²)	13	0.232	0.891	0.05	-0.03	0.45

ABI: ankle-brachial index; Δ ABI: difference between resting ABI and post-treadmill walking ABI; eGFR: estimated glomerular filtration rate.

Supplementary Table S7. Univariate linear regression analysis for native peak T1 values averaged over all skeletal calf muscle compartments with clinical markers of peripheral artery disease in matched controls.

	**Independent Variables**	***n***	**β**	**Standard Error**	**R^2**	**Adjusted r^2**	***p*-value**
Native peak T1 averaged over all calf muscle compartments (ms)	Resting ABI	19	-0.062	-77.0	0.004	-0.06	0.80
	Δ ABI	19	0.461	520.4	0.21	0.17	0.047
	Claudication onset time (sec)	N/A					
	Peak walking time (sec)	19	0.180	0.950	0.03	-0.03	0.46
	Body mass index (kg/m²)	19	0.256	5.37	0.07	0.01	0.29
	eGFR (ml/min/1.73m²)	18	0.011	0.068	0.00	-0.06	0.96

ABI: ankle-brachial index; Δ ABI: difference between resting ABI and post-treadmill walking ABI; eGFR: estimated glomerular filtration rate**.**

Supplementary Table S8. Pooled univariate linear regression analyses for native mean T1 values of skeletal calf muscle compartments with clinical markers of peripheral artery disease.

	**Independent Variables**	***n***	**β**	**Standard Error**	**R^2**	**Adjusted r^2**	***P*-value**
Mean T1 of AM (ms)	Resting ABI	37	-0.275	-73.6	0.08	0.05	0.10
	Δ ABI	36	-0.019	-9.88	<0.001	-0.03	0.91
	Claudication onset time (sec)	36	0.010	0.008	<0.001	-0.03	0.96
	Peak walking time (sec)	36	-0.109	-0.117	0.01	-0.02	0.53
	Body mass index (kg/m²)	37	0.123	1.93	0.02	-0.01	0.47
	eGFR (ml/min/1.73m²)	31	-0.105	-0.404	0.01	-0.02	0.57
Mean T1 of LM (ms)	Resting ABI	37	-0.359	-120.2	0.13	0.10	0.029
	Δ ABI	36	-0.043	-27.3	0.002	-0.03	0.80
	Claudication onset time (sec)	36	0.228	0.233	0.05	0.02	0.18
	Peak walking time (sec)	36	-0.006	-0.008	<0.001	-0.03	0.97
	Body mass index (kg/m²)	37	-0.084	-1.67	0.007	-0.02	0.62
	eGFR (ml/min/1.73m²)	31	-0.095	-0.423	0.009	-0.03	0.61
Mean T1 of DM (ms)	Resting ABI	37	-0.102	-26.2	0.01	-0.02	0.55
	Δ ABI	36	-0.111	-52.0	0.01	-0.02	0.52
	Claudication onset time (sec)	36	0.017	0.013	<0.001	-0.03	0.92
	Peak walking time (sec)	36	-0.093	-0.092	0.009	-0.02	0.59
	Body mass index (kg/m²)	37	-0.120	-1.82	0.01	-0.01	0.48
	eGFR (ml/min/1.73m²)	31	-0.052	-0.193	0.003	-0.03	0.78
Mean T1 of SM (ms)	Resting ABI	37	-0.151	-46.4	0.02	-0.005	0.37
	Δ ABI	36	0.122	70.0	0.02	-0.008	0.40
	Claudication onset time (sec)	36	0.105	0.097	0.01	-0.012	0.54
	Peak walking time (sec)	36	-0.143	-0.175	0.02	-0.008	0.40
	Body mass index (kg/m²)	37	-0.095	-1.71	0.01	-0.02	0.58
	eGFR (ml/min/1.73m²)	31	0.155	0.701	0.02	-0.01	0.41
Mean T1 of GM (ms)	Resting ABI	37	-0.073	-24.5	0.01	-0.02	0.67
	Δ ABI	36	-0.007	-4.63	<0.001	-0.03	0.97
	Claudication onset time (sec)	36	-0.058	-0.057	0.003	-0.03	0.74
	Peak walking time (sec)	36	0.087	0.113	0.008	-0.02	0.61
	Body mass index (kg/m²)	37	0.122	2.43	0.01	-0.01	0.47
	eGFR (ml/min/1.73m²)	31	0.024	0.116	0.001	-0.03	0.90

ABI: ankle-brachial index; Δ ABI: difference between resting ABI and post-treadmill walking ABI; eGFR: estimated glomerular filtration rate**.** AM: Anterior muscle group. LM: Lateral muscle group. DM: Deep muscle group. SM: Soleus muscle. GM: Gastrocnemius muscle.

Supplementary Table S9. Univariate linear regression analysis for native mean T1 values of skeletal calf muscle compartments in patients with peripheral artery disease (PAD) with clinical markers of PAD.

	Independent Variables	*n*	β	Standard Error	R^2	Adjusted r^2	*p*-value
Mean T1 of AM (ms)	Resting ABI	18	-0.457	-165.7	0.21	0.16	0.06
	Δ ABI	17	-0.069	-31.1	0.005	-0.06	0.79
	Claudication onset time (sec)	17	-0.086	-0.069	0.007	-0.06	0.74
	Peak walking time (sec)	17	-0.084	-0.077	0.007	-0.06	0.75
	Body mass index (kg/m²)	18	0.090	1.85	0.008	-0.05	0.72
	eGFR (ml/min/1.73m²)	13	-0.076	-0.304	0.006	-0.09	0.81
Mean T1 of LM (ms)	Resting ABI	18	-0.272	-123.3	0.07	0.02	0.27
	Δ ABI	17	-0.323	-181.0	0.10	0.04	0.21
	Claudication onset time (sec)	17	0.096	0.095	0.01	-0.06	0.71
	Peak walking time (sec)	17	0.163	0.188	0.03	-0.04	0.53
	Body mass index (kg/m²)	17	0.051	1.32	0.00	-0.06	0.84
	eGFR (ml/min/1.73m²)	13	-0.225	-0.966	0.05	-0.04	0.46
Mean T1 of DM (ms)	Resting ABI	18	0.004	1.30	0.00	-0.06	0.99
	Δ ABI	17	-0.435	-180.5	0.19	0.14	0.08
	Claudication onset time (sec)	17	-0.113	-0.082	0.01	-0.05	0.67
	Peak walking time (sec)	17	-0.033	-0.029	0.001	-0.07	0.90
	Body mass index (kg/m²)	18	-0.334	-6.81	0.11	0.06	0.18
	eGFR (ml/min/1.73m²)	13	-0.034	-0.136	0.001	-0.09	0.91
Mean T1 of SM (ms)	Resting ABI	18	-0.212	-79.0	0.05	-0.05	0.40
	Δ ABI	17	0.028	12.7	0.001	-0.07	0.91
	Claudication onset time (sec)	17	0.085	0.067	0.007	-0.06	0.75
	Peak walking time (sec)	17	-0.158	-0.145	0.03	-0.04	0.54
	Body mass index (kg/m²)	18	0.084	1.78	0.007	-0.06	0.74
	eGFR (ml/min/1.73m²)	13	0.227	0.974	0.05	-0.04	0.46
Mean T1 of GM (ms)	Resting ABI	18	-0.356	-128.5	0.13	0.07	0.15
	Δ ABI	17	-0.122	-49.0	0.02	-0.05	0.64
	Claudication onset time (sec)	17	-0.089	-208.7	0.08	0.02	0.27
	Peak walking time (sec)	17	0.164	0.135	0.02	-0.04	0.53
	Body mass index (kg/m²)	18	0.068	1.40	0.005	-0.06	0.79
	eGFR (ml/min/1.73m²)	13	-0.059	-0.231	0.003	-0.09	0.85

ABI: ankle-brachial index; Δ ABI: difference between resting ABI and post-treadmill walking ABI; eGFR: estimated glomerular filtration rate; ms: milliseconds**.** AM: Anterior muscle group. LM: Lateral muscle group. DM: Deep muscle group. SM: Soleus muscle. GM: Gastrocnemius muscle.

Supplementary Table S10. Univariate linear regression analyses for native mean T1 values of skeletal calf muscle compartments with clinical markers of peripheral artery disease in matched controls.

	**Independent Variables**	***n***	**β**	**Standard Error**	**R^2**	**Adjusted r^2**	***p*-value**
Mean T1 of AM (ms)	Resting ABI	19	-0.036	-27.1	0.001	-0.06	0.88
	Δ ABI	19	0.005	3.43	<0.001	-0.06	0.98
	Claudication onset time (sec)	--	--	--	--	--	--
	Peak walking time (sec)	19	-0.092	-0.296	0.01	-0.05	0.71
	Body mass index (kg/m²)	19	0.182	2.31	0.03	-0.03	0.46
	eGFR (ml/min/1.73m²)	18	-0.109	-0.409	0.01	-0.05	0.67
Mean T1 of LM (ms)	Resting ABI	19	-0.256	-225.9	0.07	0.01	0.29
	Δ ABI	19	0.327	261.9	0.11	0.06	0.17
	Claudication onset time (sec)	--	--	--	--	--	--
	Peak walking time (sec)	19	0.022	0.081	<0.001	-0.06	0.93
	Body mass index (kg/m²)	19	-0.154	-2.29	0.02	-0.03	0.53
	eGFR (ml/min/1.73m²)	18	0.112	0.491	0.01	-0.05	0.66
Mean T1 of DM (ms)	Resting ABI	19	-0.309	-214.8	0.10	0.04	0.20
	Δ ABI	19	0.427	269.5	0.18	0.14	0.07
	Claudication onset time (sec)	--	--	--	--	--	--
	Peak walking time (sec)	19	-0.108	-0.318	0.01	-0.05	0.66
	Body mass index (kg/m²)	19	0.080	0.935	0.006	-0.05	0.75
	eGFR (ml/min/1.73m²)	18	-0.062	-0.213	0.004	-0.06	0.81
Mean T1 of SM (ms)	Resting ABI	19	-0.108	-105.9	0.01	-0.05	0.66
	Δ ABI	19	0.233	206.3	0.05	0.001	0.34
	Claudication onset time (sec)	--	--	--	--	--	--
	Peak walking time (sec)	19	-0.061	-0.253	0.004	-0.06	0.80
	Body mass index (kg/m²)	19	-0.202	-3.33	0.04	-0.02	0.41
	eGFR (ml/min/1.73m²)	18	0.103	0.500	0.01	-0.05	0.68
Mean T1 of GM (ms)	Resting ABI	19	-0.091	-106.6	0.01	-0.05	0.71
	Δ ABI	19	0.143	151.5	0.02	-0.04	0.56
	Claudication onset time (sec)	--	--	--	--	--	--
	Peak walking time (sec)	19	-0.035	-0.172	0.001	-0.06	0.89
	Body mass index (kg/m²)	19	0.139	2.73	0.02	-0.04	0.57
	eGFR (ml/min/1.73m²)	18	0.043	0.254	0.002	-0.06	0.86

ABI: ankle-brachial index; Δ ABI: difference between resting ABI and post-treadmill walking ABI; eGFR: estimated glomerular filtration rate**.** AM: Anterior muscle group. LM: Lateral muscle group. DM: Deep muscle group. SM: Soleus muscle. GM: Gastrocnemius muscle.

Supplementary Table S11. Univariate linear regression analysis for ECV values averaged over all skeletal calf muscle compartments in patients with peripheral artery disease (PAD) with clinical markers of PAD.

	**Independent Variables**	***n***	**β**	**Standard Error**	**R^2**	**Adjusted r^2**	***p*-value**
Mean ECV (averaged over all calf muscle compartments) (%)	Resting ABI	16	-0.056	-1.43	0.003	-0.07	0.84
	Δ ABI	15	-0.003	-0.152	<0.001	-0.08	0.99
	Claudication onset time (sec)	15	0.200	0.011	0.04	-0.03	0.47
	Peak walking time (sec)	15	0.052	0.003	0.003	-0.07	0.85
	Body mass index (kg/m^2)	16	-0.191	-0.300	0.04	-0.03	0.48
	eGFR ($ml/min/1.73m^2$)	12	0.106	0.030	0.01	-0.09	0.74

ABI: ankle-brachial index; Δ ABI: difference between resting ABI and post-treadmill walking ABI; eGFR: estimated glomerular filtration rate**;** ECV: extracellular volume fraction.

Supplementary Table S12. Univariate linear regression analysis for ECV values averaged over all skeletal calf muscle compartments with clinical markers of peripheral artery disease in matched controls.

	**Independent Variables**	***n***	**β**	**Standard Error**	**R^2**	**Adjusted r^2**	***p*-value**
Mean ECV (averaged over all calf muscle compartments) (%)	Resting ABI	12	0.059	4.09	0.003	-0.10	0.86
	Δ ABI	12	0.048	2.62	0.002	-0.10	0.88
	Claudication onset time (sec)	N/A					
	Peak walking time (sec)	12	-0.145	-0.031	0.02	-0.08	0.65
	Body mass index (kg/m²)	12	-0.035	-0.031	0.001	-0.10	0.91
	eGFR (ml/min/1.73m²)	11	0.443	-0.071	0.07	-0.04	0.44

ABI: ankle-brachial index; Δ ABI: difference between resting ABI and post-treadmill walking ABI; eGFR: estimated glomerular filtration rate**;** ECV: extracellular volume fraction.
